# Protocol for deriving distance restraints from AlphaFold for use in solution NMR structure determination

**DOI:** 10.1016/j.xpro.2025.103988

**Published:** 2025-07-28

**Authors:** Qi-Tong Lin, Peter B. Stathopulos

**Affiliations:** 1 Department of Physiology and Pharmacology, Schulich School of Medicine and Dentistry, University of Western Ontario, London, Ontario N6A5C1, Canada

**Keywords:** structural biology, NMR

## Abstract

Artificial intelligence (AI) has revolutionized structural biology but must be applied reliably. Here, we present an approach for derivation of distance restraints from AlphaFold structure predictions to aid in automated nuclear Overhauser effect (NOE) assignment during solution NMR structure determination. We describe steps for selecting reliable AlphaFold structure predictions, determination of atom distances, and generation of high-confidence distance restraints. This protocol can expedite solution NMR structure determination, reduce NOE assignment ambiguity, enhance accuracy, enable elucidation of elusive proteins, and validate structural predictions.

For complete details on the use and execution of this protocol, please refer to Lin et al.^1^

## Highlights

• Generation and evaluation of AlphaFold structure predictions
• Creation and installation of PyMOL and ChimeraX restraint-generating plugins
• Visualization of confidently predicted atom-atom distances
• Automated creation of distance restraints for solution NMR structure determination

## Abstract (editor highlights)

Publisher’s note: Undertaking any experimental protocol requires adherence to local institutional guidelines for laboratory safety and ethics.

## Graphical abstract

**Figure:**
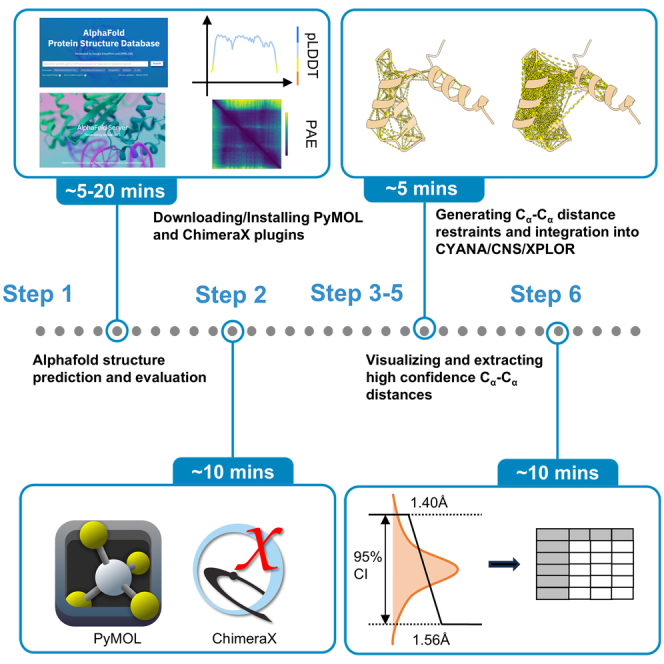

## Before you begin

**Figure 1:**
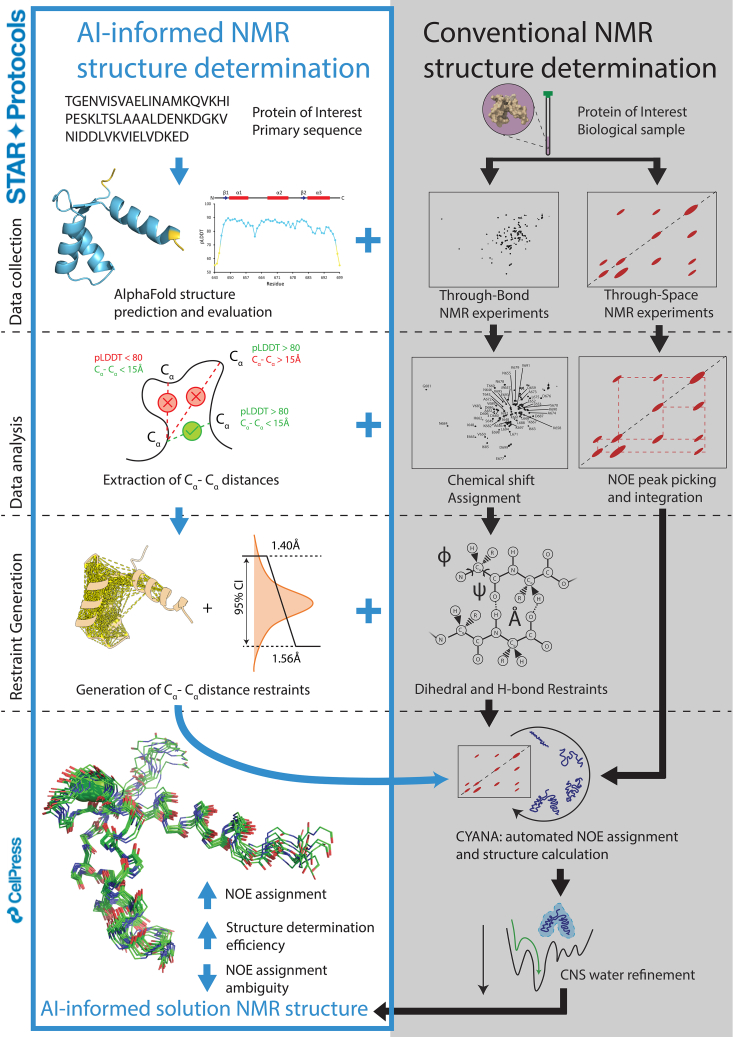
Schematic of the protocol for integrating confident AlphaFold predictions with conventional NMR structure determination

The protocol below describes the step-by-step process of extracting atom distance restraints from confident AlphaFold backbone atom predictions that can be used to inform the assignment of experimentally collected Nuclear Overhauser Effect (NOE) signals in solution NMR structure calculation. Specifically, the python-based scripts presented are compatible with PyMOL and ChimeraX, and the protocol involves the following steps: generating and evaluating the merit of AlphaFold structure predictions (step 1); downloading/creating and installing the AlphaFold ‘atom_distances’ plugin in PyMOL or ChimeraX (step 2); visualizing the atom-atom distances (C_α_-C_α_ in this test case) and generating C_α_-C_α_ distance restraint files (steps 3–5); and finally utilization/conversion of C_α_-C_α_ distance restraint files for use in CYANA/CNS/XPLOR (step 6). Using this protocol, we assigned 76% of experimentally collected and manually picked NOEs and a high-quality structural ensemble was derived that was well-defined by all the NMR data (Figure 1).^1^

AlphaFold is capable of predicting three-dimensional (3D) protein structures that rival and in some cases exceed the accuracy of solution NMR-determined structures of well-folded proteins.^2^^,^^3^^,^^4^ AlphaFold2 predicts accurate 3D models of single protein chains.^3^ AlphaFold3 expands this predictive power to include protein-protein, protein-nucleic acid, protein-ligand, protein-ion and proteins with post-translational modifications.^2^

However, there are several limitations with AlphaFold2 and AlphaFold3. Specifically, AlphaFold does not account for solvent conditions, may produce spurious stereochemical violations, struggles to predict intrinsically disordered regions accurately and lacks consistency in modeling conformational changes associated with ligand binding or unbinding.^2^ Corollary to these deficiencies, recent work has shown even very high-confidence AlphaFold predictions can differ from experimental structures on global (i.e., structural distortion and domain orientation) and local (i.e., backbone and side-chain) scales.^5^^,^^6^ Thus, while AlphaFold represents a remarkable computational advancement and is valuable for generating structural hypotheses, experimental validation remains essential for obtaining genuine insights into structural mechanisms. The inherent weakness of long-range NOE signals makes their differentiation from noise a major obstacle in solution NMR structure determination.^7^ The approach presented herein details how to leverage high-confidence AlphaFold backbone predictions to aid in assigning experimental NOEs under defined solvent conditions, highlighting a synergistic integration of AI-based modeling with solution NMR structure determination. Integrating reliable AlphaFold predictions with solution NMR methodology can significantly reduce NOE assignment ambiguity, accelerate structure determination, enhance accuracy, boost the number of assignable NOEs and enable structure resolution for proteins with sparse experimental data.

### AlphaFold structure prediction workflow

**Figure 2:**
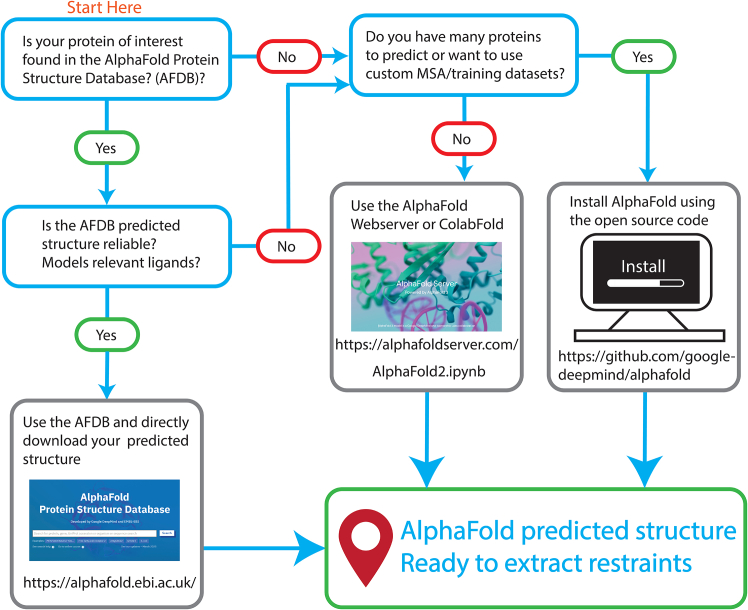
Suggested workflow and options for the implementation and use of AlphaFold in the generation of predicted protein structures

This protocol extracts high confidence atom-atom distances from predicted AlphaFold structures for use as restraints in assignment of experimentally derived NOE peaks. Hence, AlphaFold structure predictions of the proteins of interest are required. In this test case, we focus on the human Leucine zipper EF-hand containing transmembrane protein-1 (LETM1) F-EF-hand domain (residues 643–699). This protocol does not provide step-by-step instructions for running AlphaFold. Instead, we refer readers to three main options for predicting protein structures using AlphaFold2 or AlphaFold3 (Figure 2): accessing the AlphaFold Protein Structure Database (AFDB),^8^ using a cloud-based platform such as ColabFold^9^ (AlphaFold2-based) or the AlphaFold Server^10^ (AlphaFold3-based), or running AlphaFold2 or AlphaFold3 locally using the open-source code.

1. Determine if your protein is predicted in the AlphaFold Protein Structure Database.
  a. Search for your structure by protein name, UniProt accession number or protein sequence.
  b. If your protein is available, proceed to the requirements for generation of distance restraints from AlphaFold section below.
  c. If there is no available structure or you would like to generate a new structure prediction, use a cloud-based approach or install AlphaFold on your own server or workstation using the source code.

***Note:*** Predicting protein structures with cloud-based web servers allows you to generate models without local installation of the AlphaFold software. Cloud-based servers are the quickest and simplest way to perform AlphaFold structure predictions. These applications require you to fill out an online input form to run the web service. Runtime depends on the number of residues in the input sequence and server load but is typically completed within 10–15 min.

***Note:***ColabFold is a community implementation of a cloud-based Colab system for running AlphaFold2.^9^ This implementation offers control over adjustable parameters such as the depth of the Multiple Sequence Alignment (MSA) and the recycle count. Further, while AlphaFold2 uses the Many-against-Many sequence searching (MMseqs2) suite^11^ to generate rapid MSAs, ColabFold can also accept custom MSAs. Graphics Processing Unit (GPU) usage allocation is restricted to 12 h at a time per user. Additionally, depending on GPU resources allocated by Google-Colab, the maximum residue number of the prediction can range from 1000–2000.

***Note:***The AlphaFold Server is a cloud-based web service that runs AlphaFold3.^10^ This implementation allows modeling of other biological molecules including DNA, RNA, ligands and ions. A daily allowance of 20 prediction requests is permitted, each limited to 5000 tokens, where each residue, nucleotide base, ligand, ion or modification counts as 1 token.

***Note:*** Predicting protein structures using the AlphaFold open-source code allows total control over prediction parameters but requires some expertise in Linux and benefits from high performance computing resources. The source code for Alphafold2 and AlphaFold3 can be found in GitHub. Detailed instructions for installation and executing the program can be found in the ‘README.md’ file in the downloaded root directory from GitHub. AlphaFold can be installed on workstations without a GPU but will run much slower than GPU-containing computers. Maximum number of residues in any prediction is limited by available integrated or dedicated GPU memory. Once installed, to run a structure prediction, a text file containing the protein sequence in FASTA format as input is minimally needed.

***Note:*** Another option for local installations of AlphaFold is the use of virtual machines (VM) such as NMRBox,^12^ which currently offers Alphafold2 VMs free for academic users.

**CRITICAL:** The source code for Alphafold2 is available on GitHub under the Apache 2.0 License, and the model parameters are available under the Creative Commons Attribution 4.0 International License (CC BY 4.0), allowing for commercial use of the software and predictions. In contrast, while the AlphaFold3 source code is available on GitHub, the source code is licensed under the Creative Commons Attribution-NonCommercial-ShareAlike 4.0 International License (CC-BY-NC-SA 4.0), and the model parameters are subject to terms of use that explicitly prohibit commercial applications. Thus, while academic and non-profit researchers can freely use AlphaFold3 after formally requesting the model parameters from Google DeepMind, commercial entities are prohibited from doing so.

### Requirements for generation of distance restraints from AlphaFold

This protocol uses Python scripts to identify and visualize distances between atoms from AlphaFold predicted structures (i.e., .pdb or .cif file types). These scripts are applied in the form of plugins to the popular molecular viewing software PyMOL^13^ and ChimeraX.^14^^,^^15^ PyMOL and ChimeraX are downloadable at the links in the key resources table below. Both PyMOL and ChimeraX binaries are available for Windows (Windows 10 or later), macOS (12 or later) and Linux (20.04 or later) but can also be compiled from source code available at GitHub. The AlphaFold (AF) distance plugins require Python 3, which is already bundled when installing PyMOL and ChimeraX. The step-by-step protocol herein assumes either PyMOL or ChimeraX is already installed on your computer. Latest versions of PyMOL (v.3.0+) and ChimeraX (v.1.9.1) are recommended; however, any previous version utilizing Python 3 is compatible (i.e., PyMOL v.2.0+; all versions of ChimeraX).

## Key resources table

**Table:** 

REAGENT or RESOURCE	SOURCE	IDENTIFIER
**Deposited data**		

Chemical shift assignments of the human LETM1 F-EF-hand domain in the presence of calcium	Lin et al.^1^	BMRB: 31155
Solution NMR structure of the human LETM1 F-EF-hand domain in the presence of calcium	Lin et al.^1^	PDB: 9BA1

**Software and algorithms**		

Windows	Microsoft	https://www.microsoft.com/en-us/software-download/
Excel	Microsoft	https://www.microsoft.com/en-us/software-download/
macOS	Apple	https://www.apple.com/app-store/
Ubuntu	Ubuntu	https://ubuntu.com/
Python 3	Python	https://www.python.org/downloads/
ChimeraX	Pettersen et al.^14^	https://www.cgl.ucsf.edu/chimerax
PyMOL	Schrodinger, LLC^13^	https://www.pymol.org/
CYANA	Wurz et al.,^16^ Guntert^17^	https://www.las.jp/english/cyana.html
AlphaFold Protein Structure Database	DeepMind/EMBL-EBI; Varadi et al.^8^	https://alphafold.ebi.ac.uk/
ColabFold	DeepMind; Mirdita et al.^9^	https://colab.research.google.com/github/sokrypton/ColabFold/blob/main/AlphaFold2.ipynb
AlphaFold3 server	DeepMind; Eloffson^10^	https://alphafoldserver.com/
AlphaFold2	DeepMind; Jumper et al.^3^	https://github.com/google-deepmind/alphafold
AlphaFold3	DeepMind; Abramson et al.^2^	https://github.com/google-deepmind/alphafold3
NMRBox	Maciekewski et al.^12^	https://nmrbox.nmrhub.org/software
BMRB CYANA2XPLOR	Biological Magnetic Resonance Data Bank; Hoch et al.^18^	https://bmrb.io/cyana2xplor/
PDBStat	Tejero et al.^19^	https://nesgwiki.chem.buffalo.edu/index.php/PdbStat

## Step-by-step method details

### Evaluating AlphaFold-predicted structures

**Timing: 5–10 min (per structure)**

***Note:*** AlphaFold predicted structures are accompanied by predicted local distance difference test (pLDDT) values, which serve as a per-residue (AlphaFold2) or per-atom (AlphaFold3) measure of local confidence. Additionally, both AlphaFold2 and AlphaFold3 provide a predicted aligned error (PAE) as a measure of the relative distance error (in Ångströms) between any two predicted residues. For protein complexes, AlphaFold3 also outputs interchain template modeling (iTM) and interfacial predicted template modeling (ipTM) confidence scores, where values greater than 0.8 typically indicate high-quality predictions. This protocol only considers pLDDT scores.

In this step, we evaluate the per-residue measure of local confidence from the pLDDT values.

**Figure 3:**
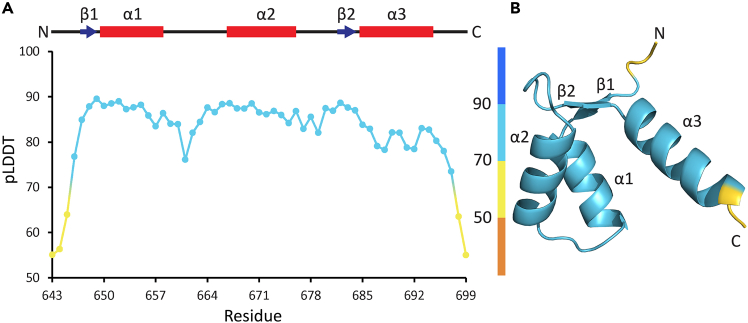
Evaluation of AlphaFold-predicted structure pLDDT score (A) Plot of Cα pLDDT score relative to residue number. This simple plot is a straightforward way to assess pLDDT score relative to motif or domain of your protein of interest. pLDDT scores can be extracted from the predicted .cif or .pdb files using any spreadsheet program. (B) Backbone cartoon representation of the predicted protein structure colored by pLDDT score (step 1). In *A and B*, the data and model shown are for the AlphaFold2-predicted human LETM1 F-EF domain (residues 643–699). In *A*, secondary structure motifs are shown above the plot with blue arrows and red cylinders representing β-strands and α-helices, respectively.

1. Visualize the pLDDT scores using one of several approaches.
  ***Note:*** Both AlphaFold2 and AlphaFold3 use the pLDDT score as a measure of confidence in the predicted structure and the metric can be treated the same in both cases. pLDDT values are scaled from 0 to 100 with higher scores indicating a more accurate prediction. pLDDT > 90 means high confidence in local backbone and side chain position; 70 < pLDDT < 90 means generally well-predicted backbone but less accurately predicted side chain positions; 50 < pLDDT < 70 means prediction may be incorrect; pLDDT < 50 means disordered or unresolved regions.
  a. Evaluate the confidence of the structure predictions using the integrated molecular visualization tools in the AlphaFold Protein Structure Database or AlphaFold Server. Protein structures are colored by their per-residue pLDDT by default.
    ***Note:*** AlphaFold-generated .pdb and .cif files contain the atom-specific coordinates of the predicted structures and the pLDDT scores on a per-residue or per-atom basis for AlphaFold2 and AlphaFold3, respectively. For both file types, the pLDDT values are stored in the B-factor column. Thus, the pLDDT scores for any atom can be viewed by opening these files in any text editor. The pLDDT scores can also be visualized over the predicted structure via ChimeraX or PyMOL, as follows.
  b. Launch PyMOL.
    i. Load the .pdb or .cif file of the predicted structure in PyMOL by selecting ‘Open’ in the ‘File’ menu (i.e., File -> Open), then navigating to and opening the desired file.
    ii. In the PyMOL command line enter:
      spectrum b, minimum=0, maximum=100
  c. Launch ChimeraX.
    i. Load the .pdb or .cif file of the predicted structure in ChimeraX by selecting ‘Open’ in the ‘File’ menu, then navigating to and opening the desired file.
    ii. In the ChimeraX command line enter:
      color bfactor palette alphafold
      ***Note:*** Per-atom pLDDT scores from AlphaFold3 predicted structures can be averaged to acquire the per-residue pLDDT scores. Averaging can be done using a spreadsheet program like Microsoft Excel. Nevertheless, the plugins provided herein will consider only the specified atom pLDDT scores (e.g., Cα if specified) when extracting atom-atom pairs (see below). There may be slight differences in the per residue pLDDT visualization compared to per-atom pLDDT but these differences should be minor. There are a variety of B-factor coloring options available in both ChimeraX and PyMOL that can be applied; we refer the reader to the PyMOL and ChimeraX wiki and usage guides for detailed instructions/options for both these programs.
  d. In ColabFold visualize the per-residue pLDDT scores found in the full array output JSON file using the ipynb notebook on the Google-Colab server.
    ***Note:*** AlphaFold uses a random “seed” to initialize modeling. This seed is automatically generated at the start of a job. Running multiple model predictions with different seeds and ranking predictions can lead to in increased prediction accuracy. In the open source AlphaFold2, AlphaFold3 and ColabFold there are numerous customizable parameters. We recommend predicting models with both AlphaFold2 and AlphaFold3, predicting full-length proteins and isolated domains and optimizing customizable parameters in your efforts to generate a model with the highest confidence scores.
    **CRITICAL:** It is important to screen structure predictions for confidence measures to ensure the C_α_-C_α_ distances extracted from the models in the next step are as reliable as possible. By default, our plugins for distance restraint generation use only C_α_ atoms with a pLDDT score > 80 to ensure the restraints are of the highest confidence. Nevertheless, backbone atoms with pLDDT scores > 70 are designated as “confident” predictions by AlphaFold, and thus, we have made the pLDDT cutoff adjustable in our scripts.
    ***Note:*** In our prediction of the human LETM1 F-EF-hand domain, no residue position in the AlphaFold2-predicted structure showed a pLDDT score > 90 that would indicate highly confident side chain positions, but 44 out of 63 residues showed pLDDT scores > 80, which should have well-predicted backbone conformations (Figure 3). No residue position in the AlphaFold3-predicted LETM1 F-EF domain structure showed a mean residue pLDDT score > 90, and only 42 out of 63 residues showed mean pLDDT scores > 80; thus, the protocol test example described below uses the AlphaFold2 human LETM1 F-EF-hand domain predicted structure to derive distance restraints.

### Generating and loading the AlphaFold distance plugins in PyMOL and ChimeraX

**Timing: 5 min**

In this step we download or create the AlphaFold distance Python script plugins for PyMOL and ChimeraX, permitting visualization of the backbone Cα-Cα distance restraints derived from the AlphaFold predicted structure.

2. Download the PyMOL and ChimeraX plugins named ‘PML_AF_generate.py’ (Data S1) and ‘CX_AF_generate.py’, (Data S2) respectively, or create the ‘.py’ plugins as follows:
  a. Copy the code (i.e., the text from the Supplemental .txt file including indents) for the preferred PyMOL (Data S3) or ChimeraX (Data S4) molecular viewer, paste into any text editor (e.g., notepad or notepad++ for Windows, VIM or gedit for Linux or TextEdit for MacOS) and save the file with a ‘.py’ extension to generate the corresponding plugin.
  b. Save the plugin in a new folder and install the plugin.
    i. For both PyMOL and ChimeraX, load the ‘PML_AF_generate.py’ and ‘CX_AF_generate.py’ plugins, respectively, by using the ‘File’ menu. Simply click ‘Open’, and select the plugin.
      ***Note:*** Since this method only temporarily loads these plugins, you must reload the plugin every time you close and restart PyMOL or ChimeraX. Alternatively, install the plugins in PyMOL and ChimeraX so they automatically load on startup.
  c. Permanently install the plugin in PyMOL using the following steps:
    i. From the ‘Plugin’ menu, select ‘Plugin Manager’.
    ii. In the ‘Install New Plugin’ tab click ‘Choose file…’, then navigate to and select the ‘PML_AF_generate.py’ file.
    iii. Click ‘Open’ to install the plugin.
    iv. Confirm that the plugin appears in the ‘Installed Plugins’ tab.
  d. Permanently install the plugin in ChimeraX using the following steps:
    i. From the ‘Favorites’ menu select ‘Settings’.
    ii. In the ‘Startup’ tab, enter the full path to the folder containing the ‘CX_AF_generate.py’ in the ‘Custom preset folder’ field.
    iii. In the same tab, enter ‘runscript CX_AF_generate.py’ in the ‘Execute these commands at startup’ field.
    iv. Click the ‘Save’ to apply the changes.
      ***Note:*** Upon successful loading, the plugins will generate the following outputs in PyMOL and ChimeraX.
      In PyMOL.
      USAGE:
      atom_distances selection1, selection2, [cutoff (default=4)],
      [contact_name (default=contacts)], [b_factor_cutoff (default=80)],
      [output_file (default=contacts.csv)]
      EXAMPLE:
      atom_distances name CA, name CA, cutoff=15, contact_name=CA_CA, b_factor_cutoff=80, output_file=PML_distances_CA_CA_15A_b80.csv
      In ChimeraX.
      Loaded CX_AF_generate.py — usage:
      atom_distances cutoff <distance> bfactor_cutoff <pLDDT> atom_name1 <atom> atom_name2 <atom>
      Example: atom_distances cutoff 15 bfactor_cutoff 80 atom_name1 CA atom_name2 CA
      executed CX_AF_generate.py

### Processing AlphaFold structure prediction coordinate file for plugin output

**Timing: 5 min**

In this step, we modify the AlphaFold predicted coordinate file residue numbering to match the native protein numbering, if necessary.

***Note:*** By default, AlphaFold starts residue numbering at 1, which may not reflect the true residue range of the protein of interest. For example, when predicted in isolation, the human LETM1 F-EF domain (residues 643–699) predicted structure we will renumber the first residue Thr from 1 to 643 to match the residue number range reflective of the wild-type, full-length protein (UniProt accession: O95202).

3. Open the coordinate file using either ChimeraX or PyMOL.
  a. In the ‘File’ menu select ‘Open’, navigate to the .cif or .pdb target file, click on the file and press ‘Open’.
  b. Renumber the starting residue sequence to match the residue number range of the wild-type, full-length protein.
    i. In PyMOL, add a number offset to all residue numbers using the following ‘alter’ command:
      alter all, resi=str(int(resi)+642)
      ***Note:*** In this command, the ‘+642’ is the positive offset and ‘all’ refers to all atoms within the coordinate file. Alternatively, you can specify a chain by replacing ‘all’ with ‘chain A’, where ‘A’ can be replaced by any chain ID.
    ii. In ChimeraX, change the starting Thr residue number from 1 to 643, using the following ‘renumber’ command:
      renumber #1 start 643
      ***Note:*** In this command, the ‘#1’ indicates the model ID, ‘643’ is the renumbered starting residue and all subsequent residues will be renumbered accordingly. Alternatively, you can specify a chain by changing ‘#1’ to ‘#1/A’, where ‘A’ can be replaced by any chain ID.
      ***Note:*** In our example, we are working with a monomer structure with a single chain. If you are working with multimers with multiple chains, make sure to accordingly and specifically renumber all chains, if necessary.

### Visualizing and extracting high-confidence atom-atom distances

**Timing: 5 min**

In this step, we use the PyMOL and ChimeraX plugins to visualize and extract from the AlphaFold predicted structure the Cα-Cα distances involving only those residues with a pLDDT score > 80.

***Note:*** The plugins allow stratification by Cα-Cα distance and pLDDT score cutoffs and automatically organize and remove symmetric duplicates.

**Figure 4:**
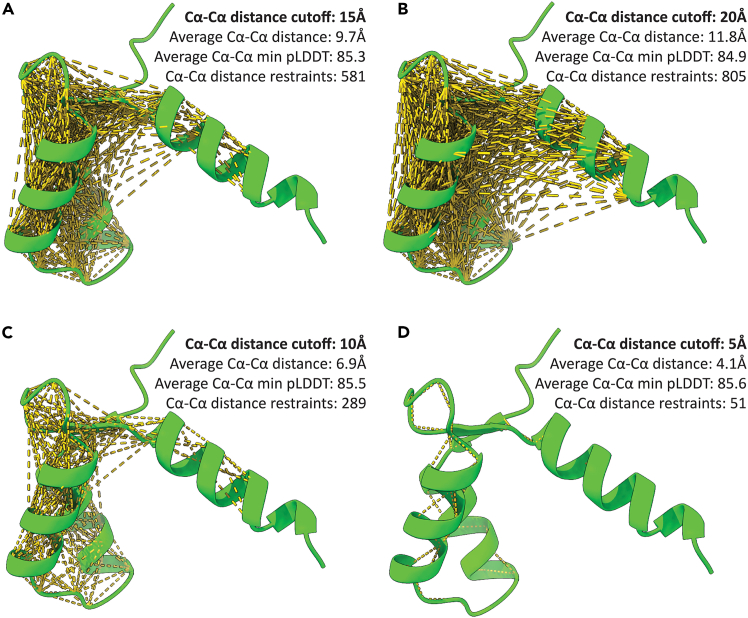
Visualization of systematic assessment of number of restraints versus distance cutoff (step 5) (A) Visualization output of the Python plugin run with a Cα-Cα atom specification, distance cutoff of 15 Å and pLDDT cutoff of 80. (B) Visualization output of the Python plugin run with a Cα-Cα atom specification, distance cutoff of 20 Å and pLDDT cutoff of 80. (C) Visualization output of the Python plugin run with a Cα-Cα atom specification, distance cutoff of 10 Å and pLDDT cutoff of 80. (D) Visualization output of the Python plugin run with a Cα-Cα atom specification, distance cutoff of 5 Å and pLDDT cutoff of 80. In *A – D*, distance restraints generated with the ChimeraX plugin are indicated with yellow dashed lines on the AlphaFold2-predicted human LETM1 F-EF domain structure (green cartoon). The PyMOL plugin visualization output is identical.

4. Set the working directory where you would like the plugin to output a master .csv file alongside processed upper and lower limit distance restraint files (i.e., .upl and .lol, respectively), ready for use in the CYANA^16^ structure calculation software.
  a. In PyMOL, click on the ‘File’ menu, then on ‘Working Directory’ and then ‘Change…’.
    i. In the file chooser window that pops up, navigate to your desired output directory and click ‘Choose’.
  b. In ChimeraX, click on the ‘File’ menu, then on ‘Set Working Folder’.
    i. In the file chooser window that pops up, navigate to your desired output directory and click ‘Choose’.
5. Visualize and extract a list of the Cα-Cα distances (or any atom-atom distance) from the AlphaFold predicted structure we previously opened in PyMOL or ChimeraX with our plugins.
  ***Note:*** To generate a list of Cα-Cα distances for only residues with a pLDDT score > 80 with distance cutoffs < 15 Å use the following script commands in PyMOL and ChimeraX:
  a. In PyMOL, run the ‘atom_distances’ command as follows (in one line and with no breaks):
    atom_distances name CA, name CA, cutoff=15, contact_name=CA_CA, b_factor_cutoff=80, output_file=PML_distances_CA_CA_15A_b80.csv
    ***Note:*** The example PyMOL command here uses ‘name CA, name CA’ to specify the atoms (i.e., Cα-Cα), ‘cutoff=15’ to specify the atom-atom distance cutoff of < 15 Å, ‘b_factor_cutoff=80’ to specify only the inclusion of atoms with pLDDT scores > 80, and ‘output_file=PML_distances_CA_CA_15A_b80.csv’ to specify the filename of the master .csv output file used to create the upper and lower limit distance restraint files. The CYANA distance restraint files will be output as ‘PML_AF_restraints.upl’ and ‘PML_AF_distances.lol’ in the same folder automatically.
  b. In ChimeraX, run the ‘atom_distances’ command as follows (in one line and with no breaks):
    atom_distances cutoff 15 bfactor_cutoff 80 atom_name1 CA atom_name2 CA
    ***Note:*** The example ChimeraX command here uses ‘cutoff 15’ to specify the atom-atom distance cutoff of < 15 Å, ‘bfactor_cutoff 80’ to specify only the inclusion of atoms with pLDDT scores > 80 and ‘atom_name1 CA atom_name2 CA’ to specify the atoms (i.e., Cα-Cα) to be used in the analysis. A master .csv output file will automatically be output with a ‘CX_distances_CA_CA_15A_b80.csv’ filename, where ‘CA_CA’, ‘15A’ and ‘b80’ will change to match the settings used in your ‘atom_distances’ command. The CYANA upper and lower distance restraint files will also be automatically outputted, named ‘CX_AF_restraints.upl’ and ‘CX_AF_distances.lol’, respectively.
    ***Note:*** The ChimeraX and PyMOL plugin ‘atom_distances’ command settings are both highly customizable. In ChimeraX, distance cutoff (i.e., cutoff), pLDDT cutoff (i.e., ‘bfactor_cutoff’) and atom name (i.e., ‘atom_name1’ and ‘atom_name2’) are adjustable parameters. In PyMOL, atom name (i.e., ‘name’), distance cutoff (i.e., ‘cutoff=’), atom-atom distance name (i.e., ‘contact_name=’), pLDDT cutoff (i.e., ‘b_factor_cutoff=’) and output .csv filename (i.e., ‘output_file=’) are adjustable parameters.
    ***Note:*** Successfully executing the PyMOL or ChimeraX ‘atom_distances’ commands will generate a visualization of the measured atom-to-atom distances in the respective molecular graphics viewers (Figure 4A). In our test case for the AlphaFold2-predicted human LETM1 F-EF domain, we found 581 unique Cα-Cα distances of < 15 Å involving only residues and Cα atoms with pLDDT scores > 80.
    ***Note:*** We found that the Cα-Cα .upl and .lol distance restraints generated with a cutoff of < 15 Å (and pLDDT scores of > 80) resulted in the most NOE assignments during structure calculation in CYANA^1^; however, the optimal distance cutoff for maximal NOE assignment may be system-dependent. Thus, it is strongly recommended to iteratively test multiple Cα-Cα distance cutoffs between 5 and 20 Å to maximize NOE assignments (Figures 4B–4D). In general, we found that increasing the distance cutoff increased longer range NOE assignments at the expense of total NOE assignments.
    **CRITICAL:** AlphaFold2 and 3 implements an inter-residue pair distance distogram that bins inter-residue pair distances from 2.31–21.68 Å with the final bin capturing all distances ≥ 21.68 Å. As such, we recommend trying multiple Cα-Cα distances cutoffs no larger than 21.68 Å.^3^

### Generation of C_α_-C_α_ distance restraints and integration into CYANA/CNS/XPLOR

**Timing: 10 min**

***Note:*** After running the ‘atom_distances’ command using either the PyMOL or ChimeraX plugins (see steps 3–5), a master .csv output file containing all the filtered and processed data is saved in your working directory alongside the .upl upper and .lol lower limit distance restraint files.

***Note:*** To generate the restraints, a gradient error factor (GEF) based on the minimum pLDDT score in the inter-residue atom pair, pLDDT cutoff and maximum pLDDT of 90 is calculated using the following equation:

$$GEF=1-\frac{{pLDDT}_{minimum}-{pLDDT}_{cutoff}}{90-{pLDDT}_{cutoff}}$$

In our example of the human LETM1 F-EF domain, we selected a pLDDT cutoff of 80, so the GEF is calculated as:

$$GEF=1-\frac{{pLDDT}_{minimum}-80}{90-80}$$

***Note:*** The GEF is subsequently used to calculate a weighted distance error in Å, using a minimum to maximum error range of 1.40–1.56 Å. This error range is derived from the 95% confidence interval of Cα RMSDs of 3,144 predicted AlphaFold2 structures compared to experimentally determined counterparts from the CASP14 dataset.^3^ The weighted error of each atom-atom distance is calculated as:

WeightedDistanceError=GEF×1.56+(1−GEF)×1.40

***Note:*** The upper and lower limit distance restraints are subsequently calculated by adding and subtracting the weighted distance error to each inter-residue atom-atom distance, respectively.

***Note:*** Using this approach, atom-atom distances that include an atom with the lowest pLDDT score (i.e., closest to the selected cutoff) are assigned the maximum distance error of 1.56 Å, while atom-atom distances where both atoms have high pLDDT scores (i.e., ≥ 90) are assigned the minimum distance error of 1.40 Å.

In this final step, we briefly describe the .upl and .lol file usage and conversion options for integration into NMR structure calculation software.

6. Retrieve the PyMOL- or ChimeraX-generated ‘PML/CX_distances.upl’ and ‘PML/CX_distances.lol’ restraint files and associated .csv files from your working directory.
  a. Open the .csv file using a spreadsheet or text editor program and visually inspect the atom-atom distances, pLDDT scores, upper distance limits and lower distance limits, ensuring they meet the criteria you specified in your ‘atom_distances’ command.
  b. If performing automated NOE assignment and structure calculation using CYANA, include the generated .upl and .lol filenames in your CALC.cya file and copy them to the appropriate path.
  c. If performing NOE assignment and structure calculation using an alternative program, convert the restraint files from CYANA format to other popular automated NOE assignment and structure calculation programs such as XPLOR-NIH,^20^ CNS^21^ and ARIA.^22^^,^^23^

***Note:*** The Biological Magnetic Resonance Data Bank (BMRB)^18^ hosts file conversion tools (https://bmrb.io/cyana2xplor/). Alternatively, PDBStat^19^ is a program developed as a universal protein NMR restraint converter, able to inter-convert between CYANA/CNS/XPLOR/ARIA/NMRStar that can be run on Linux (available here: https://github.rpi.edu/RPIBioinformatics/PDBStat_public) and can be accessed without installation through the use of VM’s already hosting PDBStat such as NMRBox.^12^

## Expected outcomes

Successful execution of these plugins will filter, visualize and extract atom-atom distances based on distance and pLDDT score cutoffs (see steps 4 and 5; Figure 4). The plugins will map the distances meeting the input criteria on the 3D structures in PyMOL or ChimeraX and output three files, all saved in the working directory set in PyMOL and ChimeraX (see step 4). The output files are described below.

**Figure 5:**
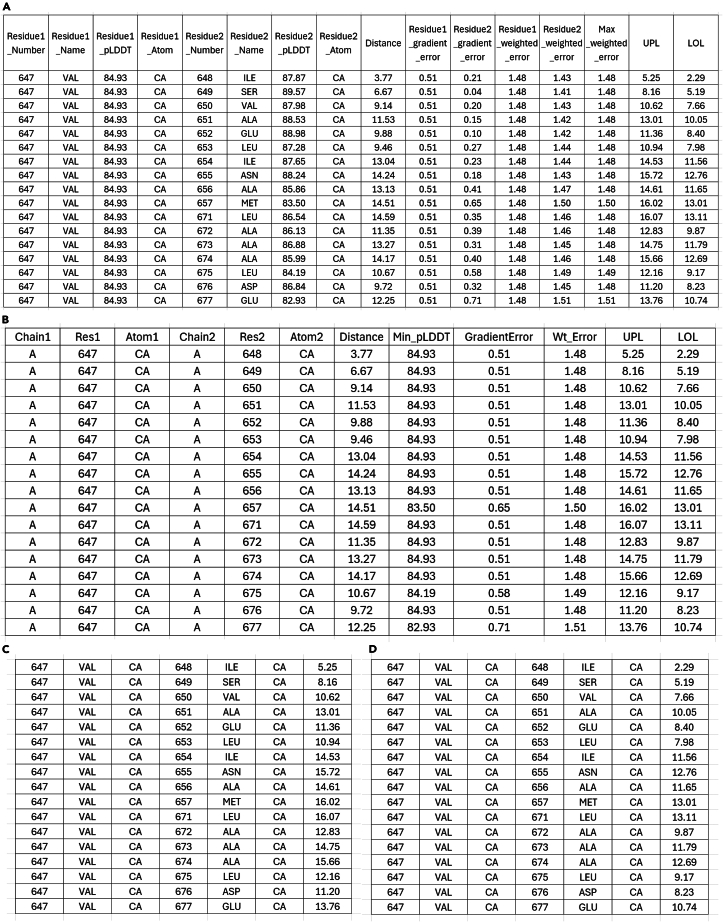
Contents of .csv,.upl and .lol files output by the Python plugin tools provided in this protocol (step 6) (A) Example master .csv table contents generated when correctly invoking the ‘atom_distances’ command using the PyMOL plugin. (B) Example master .csv table contents generated when correctly invoking the ‘atom_distances’ command using the ChimeraX plugin. (C) Example .upl file contents generated when correctly invoking the ‘atom_distances’ command using either the ChimeraX or PyMOL plugin. (D) Example .lol file contents generated when correctly invoking the ‘atom_distances’ command using either the ChimeraX or PyMOL plugin. In *A – D*, only a small portion of each of the files generated using the LETM1 F-EF structure are shown as examples.

For each atom-atom distance identified by the plugin, the PyMOL-generated master .csv includes the number of the first residue in the pair (Residue1_Number), the name of the first residue in the pair (Residue1_Name), the pLDDT score of the atom in the first residue of the pair (Residue1_pLDDT), the name of the atom in the first residue of the pair (Residue1_Atom), the number of the second residue in the pair (Residue2_Number), the name of the second residue in the pair (Residue2_Name), the pLDDT score of the atom in the second residue of the pair (Residue2_pLDDT), the name of the atom in the second residue of the pair (Residue2_Atom), the distance between the atoms (Distance), the calculated GEF for the first atom (Residue1_gradient_error), the calculated GEF for the second atom (Residue2_gradient_error), the weighted distance error for the first atom (Residue1_weighted_error), the weighted distance error for the second atom (Residue2_weighted_error), the maximum weighted error calculated from the minimum pLDDT score of the atom pair (Max_weighted_error), the upper distance limit based on the distance and maximum weighted error (UPL), and lower distance limit based on the distance and maximum weighted error (LOL). The contents of an example .csv file output using the PyMOL plugin is shown in Figure 5A.

For each atom-atom distance identified by the plugin, the ChimeraX-generated master .csv includes the chain ID of the first residue in the pair (Chain1), the residue number of the first residue in the pair (Res1), the atom name in the first residue in the pair (Atom1), the chain ID of the second residue in the pair (Chain2), the residue number of the second residue in the pair (Res2), the atom name in the second residue in the pair (Atom2), the distance between the atoms (Distance), the minimum pLDDT score in the atom pair (Min_pLDDT), the calculated GEF based on the minimum pLDDT score (GradientError), the maximum weighted distance error (Wt_Error), the upper distance limit based on the distance and maximum weighted error (UPL), and lower distance limit based on the distance and maximum weighted error (LOL). The contents of an example .csv output using the ChimeraX plugin is shown in Figure 5B.

The upper and lower limit distance restraint files ‘PML/CX_AF_restraints.upl’ and ‘PML/CX_AF_restraints.lol’ outputted by the PyMOL/ChimeraX plugins, contain the processed distance restraints in CYANA format. The CYANA .upl file has no column headers, and the data is organized such that column 1 is the residue number of the first residue in the distance pair, column 2 is the residue name of the first residue in the distance pair, column 3 is the atom name in the first residue of the distance pair, column 4 is the residue number of the second residue in the distance pair, column 5 is the residue name of the second residue in the distance pair, column 6 is atom name in the second residue of the distance pair, and column 7 is the upper distance limit for the atom-atom distance pair in Å.

The CYANA .lol file is organized in the same way except the final column contains the lower distance limit for the atom-atom distance pair in Å. The contents of the .upl and .lol output files generated using the AlphaFold2-predicted LETM1 F-EF-hand domain (residues 643–699) used in the elucidation of the solution NMR structure of this domain^1^ are shown in Figures 5C and 5D.

## Limitations

This use of restraints derived from AlphaFold structure predictions in solution NMR structure determination could introduce structural bias in the conformation. To minimize the risk of generating an incorrect structure, we recommend careful evaluation of both the local and global quality of any AlphaFold structure used in the protocol. Restraints should be derived only from highly confident backbone atom predictions, and the level of NOE assignment as well as the convergence and quality parameters of the resulting NMR structure should be critically assessed. There are both software-dependent and -independent indicators that an AlphaFold model may be inconsistent with NMR data. For the CYANA software and assuming high quality, single conformation NOE data, a discrepancy may be indicated by a high target function value for the first cycle or final cycle (i.e., > 250 and > 10 Å^2^, respectively), high ensemble RMSD for the first cycle (i.e., > 3 Å), high RMSD between the first and final cycle structures (i.e., > 3 Å), high level of discarded long-range NOEs between the first and final cycle (i.e., > 20%) and high level of unused NOEs (i.e., > 20%).^17^^,^^24^ Software-independent indicators may include steric clashes, Ramachandran outliers or sidechain outliers as well as bond-length, bond-angle, chirality, planarity, dihedral or distance violations, metrics that can be obtained from the structure ensemble using the PDB Validation Server.^25^

If the quality of the NOE data is high with relatively few artifactual peaks and the protein undergoes little or no chemical exchange, the number of assigned peaks with and without the AlphaFold restraints will be similar. In our test case, the number of assignments was only 1% lower in the absence of the AlphaFold restraints. However, the number of assignments could be improved by relaxing the chemical shift tolerances in the presence of the AlphaFold restraints. When we relaxed the proton chemical shift tolerances two-fold, we achieved 88% NOE assignment with no appreciable change in conformation and adherence to 5 of 6 CYANA-specific metrics mentioned above. If the AlphaFold-derived restraints are inconsistent with the NMR data, this may indicate a mismatch between the prediction and the experimental results. While AlphaFold2 cannot model ligand binding, AlphaFold3 allows addition of small molecules and ions in the prediction. Nevertheless, AlphaFold3 often struggles with generating accurate ligand binding/unbinding-induced conformational changes.^2^ For example, the apo LETM1 F-EF domain adopts a closed conformation in the absence of Ca^2+^ that is not accurately predicted by AlphaFold.^26^ Thus, this approach requires case-by-case evaluation of the suitability of AlphaFold structure predictions for deriving restraints to guide solution NMR structure determination.

## Troubleshooting

### Problem 1

When you copy/paste the code for the generation of your own PyMOL or ChimeraX plugin script, and the resulting file saved in your text editor cannot be opened by PyMOL or ChimeraX. Similarly, if you cannot select the newly saved plugin file or you encounter “Unrecognized file suffix ‘.txt’’ error.

### Potential solution

Ensure when saving the code in your text editor that you save the file with the file type extension ‘.py’ for python scripts (see step 2).

### Problem 2

When using the ‘atom_distances’ command in PyMOL or ChimeraX, the output .csv and distance .upl and .lol restraint files are not found.

### Potential solution

Ensure you set the working directory that indicates where you would like to save the output files (see step 4).

### Problem 3

When using the ‘atom_distances’ command in PyMOL or ChimeraX, you get an “Error writing output file: [Errno 13] Permission denied:” error message.

### Potential solution

You have either not set the working directory (see step 4) or you have set the working directory to a location requiring elevated (e.g., Administrative) permissions to write files. Check and set the working directory to a shared location accessible by all users such as your Desktop.

### Problem 4

When the AlphaFold structure prediction contains metal ions, restraints are generated in the .csv between the Ca^2+^ and Cα atoms but not for other metal ions such as K^+^, Mg^2+^ or Zn^2+^ when using the ‘atom_distances’ command and ‘CA’ atom names specified.

### Potential solution

AlphaFold3 can handle predictions with ions but labels both the residue name and atom name as the element name (e.g., the atom name for Ca^2+^ = CA, Mg^2+^ = MG, K^+^ = K, Zn^2+^ = ZN). Since the atom name for Ca^2+^ is CA, this metal ion would be captured by the ‘atom_distances’ command that involves atom names of ‘CA’. If you wish to include Cα distance restraints with non-Ca^2+^ ions you will need to explicitly name the other metal ion atom names. For example, in the case where you would like to generate Cα atom-Mg^2+^ atom restraints, the addition command in PyMOL would be:

atom_distances name CA, name MG, cutoff=15, contact_name=CA_CA, b_factor_cutoff=80, output_file=PML_distances_CA_CA_15A_b80.csv

And the ChimeraX implementation would be:

atom_distances cutoff 15 bfactor_cutoff 80 atom_name1 CA atom_name2 MG

### Problem 5

When the user wishes to use atoms other than Cα in the generation of distance restraint files.

### Potential solution

This protocol focuses on the Cα central carbon that links the carboxyl and amino groups, dictating protein backbone structure. Nevertheless, the plugin is designed such that any atom can be used in the generation of distance restraints including other backbone or side chain atoms. In the event sidechain restraints are targeted for generation, only atoms with pLDDT scores ≥ 90 should be used, and so a pLDDT cutoff of 90 should be specified when implementing the ‘atom_distances’ command (see step 5). To ensure that the GEF and weighted distance errors are properly calculated when using a pLDDT cutoff of 90, users must update the plugin scripts so that the value of ‘90’ is changed to the pLDDT maximum for the AlphaFold prediction. With this update to the scripts, the distance errors used in the .upl and .lol calculations will be a gradient between 1.40 – 1.56 Å, where the smallest error is assigned to atom pairs where both atoms have the highest pLDDT scores and the largest error is assigned to atom pairs where either atom is the closest to the pLDDT cutoff of 90. We note, however, that the 1.40–1.56 Å error range was derived the Cα RMSD of 3,144 predicted AlphaFold2 structures compared to experimentally determined counterparts,^2^ and so may not be appropriate for use in distance restraint generation between atoms other than Cα. In cases where non-protein entities are used to derive distance restraints, the user can simply modify the script(s) by replacing the 1.40 and 1.56 Å with errors they determine to be more appropriate for the molecule of interest.

## Resource availability

### Lead contact

Further information and requests for resources and reagents should be directed to and will be fulfilled by the lead contact, Peter Stathopulos (pstatho@uwo.ca).

### Technical contact

Questions about the technical specifics of performing the protocol should be directed to the technical contact, Qi-Tong Lin (qlin44@uwo.ca).

### Materials availability

This study did not generate new unique reagents.

### Data and code availability

The published article includes all code generated or analyzed during this study. We have also made the Python plugins and code publicly available at https://doi.org/10.5281/zenodo.15609023.

## Acknowledgments

This work was supported by an 10.13039/501100000038NSERC Discovery Grant (05239) to P.B.S.

## Author contributions

Q.-T.L.: conceptualization, methodology, formal analysis, investigation, writing – original draft, review and editing, and visualization. P.B.S.: conceptualization, writing – review and editing, resources, supervision, project administration, and funding acquisition.

## Declaration of interests

The authors declare no competing interests.

## Supplemental information

Data S1. “PML_AF_generate.py” PyMOL plugin, related to step 2

Data S2. “CX_AF_generate.py” ChimeraX plugin, related to step 2

Data S3. “PML_AF_generate.txt” text file of the PyMOL plugin script, related to step 2

Data S4. “CX_AF_generate.txt” text file of the ChimeraX plugin script, related to step 2
